# Risk factors associated with overweight and obesity in people with severe mental illness in South Asia: cross-sectional study in Bangladesh, India, and Pakistan

**DOI:** 10.1017/jns.2023.100

**Published:** 2023-11-21

**Authors:** Koralagamage Kavindu Appuhamy, Danielle Podmore, Alex Mitchell, Helal Uddin Ahmed, Mark Ashworth, Jan R. Boehnke, Virtu Chongtham, Asiful Haidar Chowdhury, Olga P. Garcia, Richard I. G. Holt, Rumana Huque, Krishna Prasad Muliyala, Eline Klein Onstenk, Sukanya Rajan, David Shiers, Najma Siddiqi, S. Manjunatha, Gerardo A. Zavala

**Affiliations:** 1 Department of Health Sciences, University of York, York, UK; 2 National Institute of Mental Health & Hospital, Dhaka, Bangladesh; 3 School of Lifecourse and Population Sciences, King's College, London, UK; 4 School of Health Sciences, University of Dundee, Dundee, UK; 5 Department of Psychiatry, Government Medical College and Hospital, Chandigarh, India; 6 ARK Foundation, Dhaka, Bangladesh; 7 Facultad de Ciencias Naturales, Universidad Autonoma de Queretaro, Santiago de Querétaro, Mexico; 8 Human Development and Health, Faculty of Medicine, University of Southampton, Southampton, UK; 9 Southampton National Institute for Health Research Biomedical Research Centre, University Hospital Southampton, Southampton, UK; 10 National Institute of Mental Health and Neurosciences, Bangalore, India; 11 Vrije Universiteit Amsterdam, Amsterdam, the Netherlands; 12 Psychosis Research Unit, Greater Manchester Mental Health NHS Trust, Manchester, UK; 13 Division of Psychology and Mental Health, University of Manchester, Manchester, UK; 14 School of Medicine, Keele University, Keele, UK; 15 Hull York Medical School, York, UK; 16 Bradford District Care NHS Foundation Trust, Bradford, UK

**Keywords:** Obesity, Overweight, Severe mental illness, South Asia

## Abstract

Obesity is one of the major contributors to the excess mortality seen in people with severe mental illness (SMI) and in low- and middle-income countries people with SMI may be at an even greater risk. In this study, we aimed to determine the prevalence of obesity and overweight in people with SMI and investigate the association of obesity and overweight with sociodemographic variables, other physical comorbidities, and health-risk behaviours. This was a multi-country cross-sectional survey study where data were collected from 3989 adults with SMI from three specialist mental health institutions in Bangladesh, India, and Pakistan. The prevalence of overweight and obesity was estimated using Asian BMI thresholds. Multinomial regression models were then used to explore associations between overweight and obesity with various potential determinants. There was a high prevalence of overweight (17·3 %) and obesity (46·2 %). The relative risk of having obesity (compared to normal weight) was double in women (RRR = 2·04) compared with men. Participants who met the WHO recommendations for fruit and vegetable intake had 2·53 (95 % CI: 1·65–3·88) times greater risk of having obesity compared to those not meeting them. Also, the relative risk of having obesity in people with hypertension is 69 % higher than in people without hypertension (RRR = 1·69). In conclusion, obesity is highly prevalent in SMI and associated with chronic disease. The complex relationship between diet and risk of obesity was also highlighted. People with SMI and obesity could benefit from screening for non-communicable diseases, better nutritional education, and context-appropriate lifestyle interventions.

## Introduction

Severe mental illness (SMI) is broadly defined as a group of mental disorders that are characterised by their persistence and their extensive impact on a person's life.^(1)^ This group includes schizophrenia spectrum disorders, bipolar disorder, and severe depression with psychotic features. People with SMI die on average 10–20 years earlier than the general population. Obesity and its comorbidities are common in people with SMI and are estimated to contribute to one third of the excess mortality.^(2–4)^

People with SMI tend to consume a diet low in fruits and vegetables and with more high-calorie convenience foods and sugar-sweetened beverages.^(5–7)^ This is in part due to the increased hunger caused by antipsychotics acting on various receptors.^(7)^ People with SMI also tend to be more sedentary due to negative symptoms and lack of motivation associated with their mental condition.^(8)^

The World Health Organisation (WHO) defines overweight and obesity as ‘abnormal or excessive fat accumulation that presents a risk to health’.^(9)^ Obesity is associated with a range of physical health problems including dyslipidaemia, type 2 diabetes, hypertension, cardiovascular disease, and some cancers.^(10)^ There are also psychosocial sequalae which lead to further disadvantage including lack of self-esteem and motivation, discrimination in several settings including education and employment, and a reduced quality of life.^(11)^

The global epidemic of obesity is particularly affecting people from low- and middle-income countries (LMICs) where there have been rapid changes in diet and lifestyle.^(12)^ There has been a nutrition transition away from traditional diets consisting of non-processed foods and pulses towards more energy-dense foods with added sugars and fats.^(12,13)^ Furthermore, there has been a decline in physical activity due to more sedentary jobs, and the increasing use of motorised transport.^(12)^ In addition to the high prevalence of obesity, more than 80 % of people with mental illness live in LMICs.^(14)^

The overlap between obesity and mental illness is therefore likely to be significant in South Asia where mental health service provision continues to be scarce.^(14)^ There is also an increased prevalence of diabetes and other cardiovascular risk factors seen at lower BMI values in the Asian population, as well as higher body fat found at lower BMI values.^(15,16)^ WHO guidance has, therefore, suggested the use of lower cut-off values for the Asian population based on such risk factors and comorbidities.^(17)^

Despite the increasing prevalence of SMI and obesity in LMICs, there is scarce evidence examining the scale of this comorbidity in the most affected areas; only 20 % of studies related to obesity in people with SMI have been conducted in LMICs.^(4)^ The disproportionate representation of higher-income settings means that evidence-based strategies may not take into account the societal and cultural contexts specific to LMICs.

There is an urgent need to understand the prevalence of overweight and obesity in people with SMI in LMICs to guide practice and policy and aid in the development and adaptation of targeted interventions. Determining what association there may be between specific health problems and health-risk behaviours with obesity and overweight will identify those most at risk. This study aims to (1) determine the prevalence of obesity and overweight in adults with SMI in Bangladesh, India, and Pakistan and (2) investigate the association of obesity and overweight with sociodemographic variables, physical health conditions, and health-risk behaviours.

## Methods

### Study design

This is a study based on a cross-sectional survey that has taken place across mental health institutes in Bangladesh, India, and Pakistan, looking at the physical health of people with SMI, as part of the IMPACT programme.^(18)^

### Setting

The cross-sectional survey took place across three national specialist mental health institutions: the National Institute of Mental Health and Hospital (NIMHH) in Dhaka, Bangladesh; the National Institute of Mental Health and Neurosciences (NIMHANS) in Bengaluru, India; the Institute of Psychiatry (IOP) Rawalpindi Medical University, Pakistan.^(18)^ Although they are tertiary care units, the general lack of mental health care provision for SMI at the primary and secondary care level means they serve the general population of people with SMI from across each country.

### Participants

Adults (over 18 years old) who were diagnosed with SMI by their local physician and able to provide informed consent were invited to participate in the study. SMI was defined using the International Classification of Disease 10th Revision (ICD-10) as schizophrenia, schizotypal, and delusional disorders (F20-29), bipolar affective disorder (F30, F31), and severe depression with psychotic symptoms (F32·3, F33·3). Diagnosis of SMI was confirmed by trained researchers using the Mini International Neuropsychiatric Interview (MINI) V.6.0.^(19)^

### Recruitment and consent

A total of 5801 potential participants were approached during the recruitment period, of these, 3989 adults with SMI from the three national specialist mental health institutions agreed to participate. Researchers provided written and verbal information about the study to all eligible participants, after which informed consent was sought (or, if incapacitated at the initial point of contact, sought later if capacity was regained during the study period).

We recruited inpatients and outpatients, with approximately 20 % inpatients and 80 % outpatients reflecting the usual proportions seen in practice.^(18)^ At NIMH and NIMHANS, patients were randomly selected. Due to low patient flow at IOP, all patients attending this centre during the study period were invited to participate. The recruitment of participants and data collection took place between June 2019 and January 2022.

### Data collection

Face-to-face interviews were carried out to collect information about mental and physical health, risk factors, and health-risk behaviours.^(20)^ The survey was translated into the most common local languages spoken in each country (Urdu in Pakistan, Bangla in Bangladesh, Hindi, and Kannada in India).

### Dependent variables and measurement

#### BMI categories

To calculate BMI, we measured the height and weight of all participants in accordance with the WHO guidelines.^(21)^ Height was measured to a precision of 0·1 cm using a portable height measuring board with participants removing footwear or headgear. Weight was measured in kilograms using a portable weighing scale, with participants in light clothing and no footwear. Both height and weight measurements were taken twice, and the average was used for analysis.

BMI was calculated (weight (kg)/height (m)²) and BMI categories were assigned using both international (normal weight 18·5 kg/m^2–^24·99 kg/m^2^; overweight 25 kg/m^2–^29·99 kg/m^2^; obesity ≥30 kg/m^2^) and Asian thresholds which have lower cut-offs (normal weight 18·5 kg/m^2^ 22·99 kg/m^2^; overweight 23 kg/m^2–^24·99 kg/m^2^; obesity ≥ 25 kg/m^2^).^(15,17)^

#### Abdominal obesity

To determine the abdominal obesity, waist circumference was measured in duplicate to a precision of 0·1 cm using flexible fibreglass tape at the end of normal expiration, between the lower margin of the last palpable rib and the top of the iliac crest.^(21)^ Ethnicity-specific cut-off values for waist circumference have been recommended for the Asian population by International Diabetes Federation (IDF): ≥90 cm for men and ≥80 cm for women.^(22)^

### Independent variables and measurement

Several factors associated with obesity were investigated using the WHO STEPS instrument version 3.2,^(20)^ including physical comorbidities, health-risk behaviours, and sociodemographic variables.

#### Physical comorbidities

Hypertension was defined by blood pressure (BP) exceeding the cut-off (Systolic BP >140 mmHg or Diastolic BP >90 mmHg) when measured during the survey, or those who reported diagnosis from a healthcare professional. BP was measured according to the WHO guidelines,^(21)^ using an automated BP monitor (OMRON).

Type 2 diabetes was defined by the HbA_1c_ measurement ≥6·5 % (48 mmol/mol) and those who self-reported. We also defined pre-diabetes according to the American Diabetes Association,^(23)^ as HbA_1c_ between 5·7 to 6·4 % (39–47 mmol/mol).

High cholesterol was defined as a low-denisty lipoprotein (LDL) concentration ≥1 g/l according to their serum test during the survey and those who self-reported.^(24)^ High triglycerides were defined solely based on serum blood tests (≥1.8 g/l).^(24)^ All blood collection was carried out in accordance with the WHO STEPS surveillance manual.^(21)^

#### Health-risk behaviours

Variables were based upon whether the participants followed WHO recommendations for physical activity and fruit and vegetable intake.^(25)^ Self-reported current smoking status was also recorded.

#### Sociodemographic and clinical variables

The variables included SMI diagnosis, SMI duration (years), antipsychotic use, clinical setting (inpatient/outpatient), and sociodemographic variables; age, sex, highest level of education, work status, and income tertiles.

### Sample size

A sample size of 865 was originally calculated to be able to estimate the prevalence of diabetes with a precision of 2 % as an example of survey precision. However, this sample size is also sufficient to estimate the prevalence of obesity with a precision <2 %, considering a prevalence estimate of 10 %.^(18)^

### Statistical analysis

This study was reported according to STROBE guidelines.^(26)^ All statistical analyses were carried out using Stata v.17. Statistical significance was assessed at the 5 % level.

Participant characteristics were summarised descriptively for each country and overall. Continuous variables were reported as means and standard deviations (and/or median, interquartile range, and minimum and maximum as appropriate), and categorical variables were reported as frequencies and percentages.

For each country separately and overall, the prevalence of underweight, normal weight, overweight, and obesity was reported using both the WHO international and the Asian BMI cut-off values. The prevalence of normal weight, overweight and obesity, using Asian cut-off values, was stratified by key characteristics (sex, age, SMI diagnosis and setting), reported by country and overall.

To investigate the associations between BMI categories and other comorbidities, multinomial logistic regression models were fitted with BMI group as the dependent variable. In the interests of accurately quantifying the disease burden of obesity and ensuring it is clinically relevant for the target population, Asian cut-off values were used.

Individuals classified as underweight, according to BMI category, were excluded from this analysis as the examination of the association of being underweight with associated risk factors was not an objective of this study. Physical health comorbidities (diabetes, hypertension, high cholesterol, high triglycerides), health-risk behaviours (smoking status, physical activity, diet), and sociodemographic variables (age group, sex, type of SMI, SMI duration, antipsychotic medication, setting, highest level of education, employment status, income tertile, and country) were included as independent variables. Interactions between the independent variables and country were assessed using a likelihood ratio test to compare these to the model with no interaction terms. Relative risk ratios (RRR) were reported along with corresponding 95 % confidence intervals and *P*-values. Unadjusted estimates are reported in the appendix.

The associations between abdominal obesity and its determinants were investigated. Logistic regression models were fitted with abdominal obesity as the dichotomous (yes/no) dependent variable. The same variables as in the analysis of BMI categories were included as independent variables. Odds ratios (OR) were reported along with corresponding 95 % confidence intervals and *P*-values. Unadjusted estimates are reported in the appendix.

Analysis models included complete cases only; however, multiple imputation (MI) was performed as a sensitivity analysis, using chained equations to impute missing data. The results were not changed by the MI analysis. Results of the MI analyses are presented in the appendix alongside the complete-case analysis for comparison.

## Results

From the 3989 participants included in the study, 3126 (78·4 %) participants were included in the multinomial logistic regression (BMI categories), and 3389 participants (85·0 %) were included in the binomial logistic regression (abdominal obesity). The excluded participants for each analysis, and the reasons for this, are detailed in the flowchart found in the appendix.

### Participant characteristics

The characteristics of study participants are presented in Table 1. A total of 59·1 % of the participants were male. The average age was 35·8 years (sd 11·9; range 18–84 years). Schizophrenia-type disorder was the most common type of SMI with 44·7 % of participants having this diagnosis. The majority of the sample were outpatients (82·5 %), and the lowest income group was the most common (41·4 %).

**Table 1.** Participant characteristics summarised overall and by country

	Bangladesh (*n* 1500)	India (*n* 1175)	Pakistan (*n* 1314)	Overall (*n* 3989)
Sex, *n* (%)				
Number with data	1500 (100)	1175 (100)	1314 (100)	3989 (100)
Male	915 (61·0)	648 (55·1)	796 (60·6)	2359 (59·1)
Female	585 (39·0)	527 (44·9)	518 (39·4)	1630 (40·9)
Age (years)				
*n* (%)	1500 (100)	1175 (100)	1314 (100)	3989 (100)
Mean (sd)	31·5 (10·8)	38·8 (11·2)	38·1 (12·3)	35·8 (11·9)
Median (IQR)	30·0 (23·0–38·0)	38·0 (30·0–46·0)	36·0 (28·0–45·0)	35·0 (26·0–44·0)
Min, Max	18·0, 76·0	18·0, 81·0	18·0, 84·0	18·0, 84·0
Age group, *n* (%)				
Number with data	1500 (100)	1175 (100)	1314 (100)	3989 (100)
18–24 years	434 (28·9)	123 (10·5)	159 (12·1)	716 (17·9)
25–39 years	732 (48·8)	538 (45·8)	603 (45·9)	1873 (47·0)
40–54 years	263 (17·5)	386 (32·9)	402 (30·6)	1051 (26·3)
55+ years	71 (4·7)	128 (10·9)	150 (11·4)	349 (8·7)
SMI, *n* (%)				
Number with data	1500 (100)	1175 (100)	1314 (100)	3989 (100)
Schizophrenia-type disorder	935 (62·3)	673 (57·3)	176 (13·4)	1784 (44·7)
Major depressive disorder with psychotic features	77 (5·1)	63 (5·4)	601 (45·7)	741 (18·6)
Bipolar disorder	488 (32·5)	439 (37·4)	537 (40·9)	1464 (36·7)
Duration of the SMI, *n* (%)				
Number with data	1500 (100)	1175 (100)	1314 (100)	3989 (100)
≤2 years	436 (29·1)	215 (18·3)	289 (22·0)	940 (23·6)
3–5 years	457 (30·5)	266 (22·6)	320 (24·4)	1043 (26·1)
6–10 years	332 (22·1)	299 (25·4)	299 (22·8)	930 (23·3)
>10 years	271 (18·1)	359 (30·6)	399 (30·4)	1029 (25·8)
Do not know/Don't remember	4 (0·3)	36 (3·1)	7 (0·5)	47 (1·2)
Setting, *n* (%)				
Number with data	1500 (100)	1175 (100)	1314 (100)	3989 (100)
Inpatient	313 (20·9)	264 (22·5)	122 (9·3)	699 (17·5)
Outpatient	1187 (79·1)	911 (77·5)	1192 (90·7)	3290 (82·5)
Highest level of education, *n* (%)				
Number with data	1500 (100)	1174 (99·9)	1312 (99·8)	3986 (99·9)
No formal education	151 (10·1)	141 (12·0)	257 (19·6)	549 (13·8)
Primary education	842 (56·1)	401 (34·2)	234 (17·8)	1477 (37·1)
Secondary education	234 (15·6)	228 (19·4)	273 (20·8)	735 (18·4)
Higher/more than secondary	273 (18·2)	404 (34·4)	548 (41·8)	1225 (30·7)
Work status (past 12 months), *n* (%)				
Number with data	1500 (100)	1174 (99·9)	1309 (99·6)	3983 (99·8)
Employed	439 (29·3)	522 (44·5)	507 (38·7)	1468 (36·9)
Unemployed	595 (39·7)	227 (19·3)	291 (22·2)	1113 (27·9)
Other^a^	466 (31·1)	425 (36·2)	511 (39·0)	1402 (35·2)
Monthly household income (USD)				
*n* (%)	1497 (99·8)	1032 (87·8)	1307 (99·5)	3836 (96·2)
Mean (sd)	224·6 (352·1)	305·2 (861·4)	198·6 (199·8)	237·4 (513·1)
Median (IQR)	176·7 (117·8–235·5)	158·9 (66·2–264·8)	148·6 (89·2–237·8)	158·9 (105·9–264·8)
Min, Max	11·8, 11,777·2	0·0, 16,551·9	0·0, 2972·7	0·0, 16,551·9
Income tertile, *n* (%)				
Number with data	1497 (99·8)	1032 (87·8)	1307 (99·5)	3836 (96·2)
Low	674 (45·0)	349 (33·8)	566 (43·3)	1589 (41·4)
Middle	485 (32·4)	463 (44·9)	315 (24·1)	1263 (32·9)
High	338 (22·6)	220 (21·3)	426 (32·6)	984 (25·7)
Marital status, *n* (%)				
Number with data	1500 (100)	1175 (100)	1314 (100)	3989 (100)
Never married	539 (35·9)	349 (29·7)	417 (31·7)	1305 (32·7)
Currently married	818 (54·5)	711 (60·5)	747 (56·8)	2276 (57·1)
Ever married^b^	143 (9·5)	115 (9·8)	150 (11·4)	408 (10·2)
BMI				
*n* (%)	1497 (99·8)	1161 (98·8)	1304 (99·2)	3962 (99·3)
Mean (sd)	24·0 (4·5)	25·6 (5·3)	25·9 (6·1)	25·1 (5·4)
Median (IQR)	23·6 (21·1–26·5)	25·0 (22·1–28·5)	25·3 (21·8–29·3)	24·5 (21·6–28·1)
Min, Max	14·0, 83·8	13·0, 96·9	9·7, 65·5	9·7, 96·9
BMI (WHO international classification), *n* (%)				
Number with data	1497 (99·8)	1161 (98·8)	1304 (99·2)	3962 (99·3)
Underweight (BMI <18·5)	129 (8·6)	66 (5·7)	113 (8·7)	308 (7·8)
Normal weight (BMI 18·5–24·9)	817 (54·6)	506 (43·6)	501 (38·4)	1824 (46·0)
Overweight (BMI 25·0–29·9)	405 (27·1)	384 (33·1)	406 (31·1)	1195 (30·2)
Obesity (BMI ≥30·0)	146 (9·8)	205 (17·7)	284 (21·8)	635 (16·0)
BMI (WHO Asian cut-offs), *n* (%)				
Number with data	1497 (99·8)	1161 (98·8)	1304 (99·2)	3962 (99·3)
Underweight (BMI <18·5)	129 (8·6)	66 (5·7)	113 (8·7)	308 (7·8)
Normal weight (BMI 18·5–22·9)	523 (34·9)	290 (25·0)	327 (25·1)	1140 (28·8)
Overweight (BMI 23·0–24·9)	294 (19·6)	216 (18·6)	174 (13·3)	684 (17·3)
Obesity (BMI ≥25·0)	551 (36·8)	589 (50·7)	690 (52·9)	1830 (46·2)
Waist circumference (cm)				
Males				
*n* (% of males)	915 (100)	640 (98·8)	786 (98·7)	2341 (99·2)
Mean (sd)	83·3 (11·2)	90·3 (12·3)	89·9 (16·8)	87·4 (14·0)
Median (IQR)	84·5 (75·8–90·5)	90·0 (82·0–98·0)	89·2 (81·0–99·0)	87·5 (79·2–95·0)
Min, Max	35·5, 163·3	50·6, 140·0	30·3, 191·2	30·3, 191·2
Females				
*n* (% of females)	582 (99·5)	516 (97·9)	507 (97·9)	1605 (98·5)
Mean (sd)	84·3 (12·2)	87·2 (13·2)	90·6 (22·2)	87·2 (16·5)
Median (IQR)	85·5 (76·5–91·5)	87·5 (79·0–95·3)	92·0 (78·2–105·0)	87·6 (78·0–96·5)
Min, Max	46·5, 174·5	40·0, 130·2	30·0, 193·2	30·0, 193·2
Abdominal obesity, *n* (%)				
Males				
Number with data	915 (100)	640 (98·8)	786 (98·7)	2341 (99·2)
No abdominal obesity	666 (72·8)	300 (46·9)	398 (50·4)	1364 (58·3)
Has abdominal obesity	249 (27·2)	340 (53·1)	388 (49·4)	977 (41·7)
Females				
Number with data	582 (99·5)	516 (97·9)	507 (97·9)	1605 (98·5)
No abdominal obesity	189 (32·5)	138 (26·7)	133 (26·2)	460 (28·7)
Has abdominal obesity	393 (67·5)	378 (73·3)	374 (73·8)	1145 (71·3)
Males and Females				
Number with data	1497 (99·8)	1156 (98·4)	1293 (98·4)	3946 (98·9)
No abdominal obesity	855 (57·1)	438 (37·9)	531 (41·1)	1824 (46·2)
Has abdominal obesity	642 (42·9)	718 (62·1)	762 (58·9)	2122 (53·8)

a Other includes: homemaker, student, and retired.

b Ever married includes: widowed, separated, and divorced. sd, Standard deviation. IQR, Interquartile range.

### Prevalence of obesity and overweight

The overall prevalence of obesity across the three countries was 16·0 % according to the WHO international BMI cut-offs and 46·2 % according to the WHO Asian cut-offs. The overall prevalence of overweight across the three countries was 30·2 % according to WHO international BMI cut-offs and 17·3 % according to Asian cut-offs.

The overall prevalence of abdominal obesity was 53·8 %; however, differences were observed between sexes and countries. Abdominal obesity was less prevalent in men from Bangladesh (27·2 %) compared to India (53·1 %) and Pakistan (49·4 %) despite relatively similar prevalence found amongst the female participants in each country.

### Overall and stratified prevalence of BMI categories

According to the Asian cut-offs, the largest proportion of participants (46·2 %) were classified as having obesity, compared to the international cut-offs, where the largest proportion (46·0 %) were classed as having normal weight (Table 2).

**Table 2.** Overall prevalence (using WHO international and WHO Asian cut-offs) and stratified prevalence (using WHO Asian cut-offs) of normal weight, overweight and obesity for all countries (Bangladesh, India, Pakistan)

	All countries (*n* 3962)		
Frequency prevalence (95 % CI)	Prevalence of normal weight	Prevalence of overweight	Prevalence of obesity
Overall (WHO Asian cut-offs)	1140/3962	684/3962	1830/3962
	28·8 (27·4–30·2)	17·3 (16·1–18·5)	46·2 (44·6–47·7)
Overall (WHO international cut-offs)	1824/3962	1195/3962	635/3962
	46·0 (44·5–47·6)	30·2 (28·8–31·6)	16·0 (14·9–17·2)
Stratified prevalence (WHO Asian cut-offs):			
Sex			
Male	739/2349	460/2349	958/2349
	31·5 (29·6–33·4)	19·6 (18·0–21·2)	40·8 (38·8–42·8)
Female	401/1613	224/1613	872/1613
	24·9 (22·8–27·0)	13·9 (12·3–15·7)	54·1 (51·6–56·5)
Age group			
18–24 years	325/710	113/710	188/710
	45·8 (42·1–49·5)	15·9 (13·4–18·8)	26·5 (23·4–29·8)
25–39 years	497/1861	339/1861	891/1861
	26·7 (24·7–28·8)	18·2 (16·5–20·0)	47·9 (45·6–50·2)
40–54 years	224/1044	172/1044	585/1044
	21·5 (19·1–24·1)	16·5 (14·4–18·9)	56·0 (53·0–59·0)
55+ years	94/347	60/347	166/347
	27·1 (22·7–32·0)	17·3 (13·7–21·6)	47·8 (42·6–53·1)
SMI			
Bipolar disorder (any)	387/1455	244/1455	742/1455
	26·6 (24·4–28·9)	16·8 (14·9–18·8)	51·0 (48·4–53·6)
Major depressive disorder with psychotic features	189/734	103/734	378/734
	25·7 (22·7–29·0)	14·0 (11·7–16·7)	51·5 (47·9–55·1)
Schizophrenia-type disorder	564/1773	337/1773	710/1773
	31·8 (29·7–34·0)	19·0 (17·2–20·9)	40·0 (37·8–42·3)
Setting			
Inpatient	259/686	113/686	257/686
	37·8 (34·2–41·4)	16·5 (13·9–19·4)	37·5 (33·9–41·2)
Outpatient	881/3276	571/3276	1573/3276
	26·9 (25·4–28·4)	17·4 (16·2–18·8)	48·0 (46·3–49·7)

For participants where BMI was calculable (*n =* 3962).

### Overall and stratified prevalence for each country

The prevalence of obesity in India and Pakistan was similar (50·7 % [95 % CI: 47·9–53·6] and 52·9 % [95 % CI: 50·2–55·6] respectively), and higher than in Bangladesh (36·8 % [95 % CI: 34·4–39·3]) (Table 3). Overall, the proportion of men with normal weight (31·5 % [95 % CI: 29·6–33·4]) was greater than the proportion of women with normal weight (24·9 % [95 % CI: 22·8–27·0]). Obesity was most prevalent in the 40–54-year age group (56·0 % [95 % CI: 53·0–59·0]) and least prevalent amongst 18–24-year-olds (26·5 % [95 % CI: 23·4–29·8]). Obesity was most prevalent in those with a diagnosis of major depressive disorder with psychotic symptoms in Bangladesh (43·4 % [95 % CI: 32·8–54·7]), and most prevalent in people with bipolar disorder in India (56·7 % [95 % CI: 52·0–61·3]) and Pakistan (57·3 % [95 % CI: 53·1–61·4]). In addition, a greater proportion of outpatients had obesity (48 % [95 % CI: 46·3–49·7]) compared to the inpatients (37·5 % [95 % CI: 33·9–41·2]). The prevalence of underweight was 8·6 % (95 % CI: 7·3–10·2) for Bangladesh, 8·7 % (95 % CI: 7·3–10·3) for Pakistan, and 5·7 % (95 % CI: 4·5–7·2) for India.

**Table 3.** Overall prevalence (using WHO international and WHO Asian cut-offs) and stratified prevalence (using WHO Asian cut-offs) of normal weight, overweight and obesity in Bangladesh, India, Pakistan^a^

	Bangladesh (*n* 1497)			India (*n* 1161)			Pakistan (*n* 1304)		
Frequency prevalence (95 % CI)	Prevalence of normal weight	Prevalence of overweight	Prevalence of obesity	Prevalence of normal weight	Prevalence of overweight	Prevalence of obesity	Prevalence of normal weight	Prevalence of overweight	Prevalence of obesity
Overall (WHO international cut-offs)	817/1497	405/1497	146/1497	506/1161	384/1161	205/1161	501/1304	406/1304	284/1304
	54·6 (52·0–57·1)	27·1 (24·9–29·4)	9·8 (8·4–11·4)	43·6 (40·8–46·5)	33·1 (30·4–35·8)	17·7 (15·6–20·0)	38·4 (35·8–41·1)	31·1 (28·7–33·7)	21·8 (19·6–24·1)
Overall (WHO Asian cut-offs)	523/1497	294/1497	551/1497	290/1161	216/1161	589/1161	327/1304	174/1304	690/1304
	34·9 (32·6–37·4)	19·6 (17·7–21·7)	36·8 (34·4–39·3)	25·0 (22·6–27·6)	18·6 (16·5–21·0)	50·7 (47·9–53·6)	25·1 (22·8–27·5)	13·3 (11·6–15·3)	52·9 (50·2–55·6)
Stratified prevalence (WHO Asian cut-offs):									
Sex									
Male	336/915	190/915	300/915	175/643	145/643	280/643	228/791	125/791	378/791
	36·7 (33·7–39·9)	20·8 (18·3–23·5)	32·8 (29·8–35·9)	27·2 (23·9–30·8)	22·6 (19·5–25·9)	43·5 (39·8–47·4)	28·8 (25·8–32·1)	15·8 (13·4–18·5)	47·8 (44·3–51·3)
Female	187/582	104/582	251/582	115/518	71/518	309/518	99/513	49/513	312/513
	32·1 (28·5–36·0)	17·9 (15·0–21·2)	43·1 (39·2–47·2)	22·2 (18·8–26·0)	13·7 (11·0–16·9)	59·7 (55·4–63·8)	19·3 (16·1–22·9)	9·6 (7·3–12·4)	60·8 (56·5–65·0)
Age group									
18–24 years	196/432	76/432	113/432	53/120	17/120	38/120	76/158	20/158	37/158
	45·4 (40·7–50·1)	17·6 (14·3–21·5)	26·2 (22·2–30·5)	44·2 (35·6–53·1)	14·2 (9·0–21·6)	31·7 (24·0–40·5)	48·1 (40·4–55·9)	12·7 (8·3–18·8)	23·4 (17·5–30·6)
25–39 years	232/731	147/731	296/731	109/531	101/531	294/531	156/599	91/599	301/599
	31·7 (28·5–35·2)	20·1 (17·4–23·2)	40·5 (37·0–44·1)	20·5 (17·3–24·2)	19·0 (15·9–22·6)	55·4 (51·1–59·5)	26·0 (22·7–29·7)	15·2 (12·5–18·3)	50·3 (46·3–54·2)
40–54 years	71/263	56/263	119/263	90/382	72/382	195/382	63/399	44/399	271/399
	27·0 (22·0–32·7)	21·3 (16·8–26·7)	45·2 (39·3–51·3)	23·6 (19·6–28·1)	18·8 (15·2–23·1)	51·0 (46·0–56·0)	15·8 (12·5–19·7)	11·0 (8·3–14·5)	67·9 (63·2–72·3)
55+ years	24/71	15/71	23/71	38/128	26/128	62/128	32/148	19/148	81/148
	33·8 (23·8–45·5)	21·1 (13·2–32·1)	32·4 (22·6–44·1)	29·7 (22·4–38·2)	20·3 (14·2–28·2)	48·4 (39·9–57·1)	21·6 (15·7–29·0)	12·8 (8·3–19·3)	54·7 (46·7–62·6)
SMI									
Bipolar disorder (any)	164/487	101/487	190/487	103/434	70/434	246/434	120/534	73/534	306/534
	33·7 (29·6–38·0)	20·7 (17·4–24·6)	39·0 (34·8–43·4)	23·7 (20·0–28·0)	16·1 (13·0–19·9)	56·7 (52·0–61·3)	22·5 (19·1–26·2)	13·7 (11·0–16·9)	57·3 (53·1–61·4)
Major depressive disorder with psychotic features	22/76	17/76	33/76	12/63	17/63	34/63	155/595	69/595	311/595
	28·9 (19·9–40·1)	22·4 (14·4–33·1)	43·4 (32·8–54·7)	19·0 (11·1–30·6)	27·0 (17·5–39·2)	54·0 (41·7–65·8)	26·1 (22·7–29·7)	11·6 (9·3–14·4)	52·3 (48·2–56·3)
Psychotic disorder	337/934	176/934	328/934	175/664	129/664	309/664	52/175	32/175	73/175
	36·1 (33·1–39·2)	18·8 (16·5–21·5)	35·1 (32·1–38·2)	26·4 (23·1–29·8)	19·4 (16·6–22·6)	46·5 (42·8–50·3)	29·7 (23·4–36·9)	18·3 (13·2–24·7)	41·7 (34·6–49·2)
Setting									
Inpatient	147/311	59/311	80/311	72/255	41/255	119/255	40/120	13/120	58/120
	47·3 (41·8–52·8)	19·0 (15·0–23·7)	25·7 (21·2–30·9)	28·2 (23·0–34·1)	16·1 (12·1–21·1)	46·7 (40·6–52·8)	33·3 (25·5–42·2)	10·8 (6·4–17·8)	48·3 (39·5–57·2)
Outpatient	376/1186	235/1186	471/1186	218/906	175/906	470/906	287/1184	161/1184	632/1184
	31·7 (29·1–34·4)	19·8 (17·6–22·2)	39·7 (37·0–42·5)	24·1 (21·4–27·0)	19·3 (16·9–22·0)	51·9 (48·6–55·1)	24·2 (21·9–26·8)	13·6 (11·8–15·7)	53·4 (50·5–56·2)

a For participants where BMI was calculable (*n =* 3962 in total).

### Association of predictors of overweight and obesity

The multinomial logistic regression analysis for the association of predictors of overweight and obesity are shown in Table 4a (sociodemographic variables) and Table 4b (comorbidities and health-risk behaviours).

**Table 4a.** Adjusted associations of sociodemographic variables with overweight and obesity, using WHO Asian cut-offs

	Normal weight^b^ (*n* 1017)	Overweight^b^ (*n* 566)	Obesity^b^ (*n* 1543)	Overweight *v.* normal weight RRR (95 % CI) *P*-value	Obese *v.* normal weight RRR (95 % CI) *P*-value
Sex					
Male	668/1860 (35·9)	380/1860 (20·4)	812/1860 (43·7)	Reference	Reference
Female	349/1266 (27·6)	186/1266 (14·7)	731/1266 (57·7)	1·09 (0·78–1·51) *P*=0·611	2·04 (1·56–2·67) *P* < 0·001
Age group					
18–24 years	299/567 (52·7)	100/567 (17·6)	168/567 (29·6)	Reference	Reference
25–39 years	452/1484 (30·5)	284/1484 (19·1)	748/1484 (50·4)	1·73 (1·29–2·32) *P* < 0·001	2·36 (1·84–3·02) *P* < 0·001
40–54 years	188/811 (23·2)	138/811 (17·0)	485/811 (59·8)	2·05 (1·43–2·95) *P* < 0·001	2·91 (2·15–3·92) *P* < 0·001
55+ years	78/264 (29·5)	44/264 (16·7)	142/264 (53·8)	1·59 (0·96–2·61) *P* = 0·070	1·63 (1·09–2·44) *P* = 0·018
SMI diagnosis					
Bipolar disorder	346/1171 (29·5)	201/1171 (17·2)	624/1171 (53·3)	Reference	Reference
Major depressive disorder with psychotic features	180/609 (29·6)	91/609 (14·9)	338/609 (55·5)	0·95 (0·67–1·34) *P* = 0·755	0·81 (0·63–1·06) *P* = 0·130
Schizophrenia-type disorder	491/1346 (36·5)	274/1346 (20·4)	581/1346 (43·2)	0·91 (0·71–1·17) *P* = 0·462	0·69 (0·57–0·85) *P* < 0·001
SMI duration					
<2 years	297/720 (41·3)	120/720 (16·7)	303/720 (42·1)	Reference	Reference
3–5 years	296/832 (35·6)	170/832 (20·4)	366/8s32 (44·0)	1·32 (0·99–1·77) *P* = 0·062	1·14 (0·90–1·45) *P* = 0·285
6–10 years	232/755 (30·7)	146/755 (19·3)	377/755 (49·9)	1·33 (0·97–1·81) *P* = 0·076	1·30 (1·01–1·68) *P* = 0·044
>10 years	192/819 (23·4)	130/819 (15·9)	497/819 (60·7)	1·36 (0·97–1·91) *P* = 0·077	1·76 (1·35–2·31) *P* < 0·001
Antipsychotic medication					
No	42/90 (46·7)	15/90 (16·7)	33/90 (36·7)	Reference	Reference
Yes	975/3036 (32·1)	551/3036 (18·1)	1510/3036 (49·7)	1·36 (0·73–2·51) *P* = 0·333	1·93 (1·17–3·19) *P* = 0·010
Setting					
Inpatient	212/486 (43·6)	84/486 (17·3)	190/486 (39·1)	Reference	Reference
Outpatient	805/2640 (30·5)	482/2640 (18·3)	1353/2640 (51·3)	1·42 (1·07–1·90) *P* = 0·017	1·57 (1·24–2·00) *P* < 0·001
Level of education					
No formal education	132/423 (31·2)	71/423 (16·8)	220/423 (52·0)	Reference	Reference
Primary education	408/1173 (34·8)	239/1173 (20·4)	526/1173 (44·8)	1·15 (0·81–1·64) *P* = 0·437	1·23 (0·92–1·65) *P* = 0·154
Secondary education	183/578 (31·7)	100/578 (17·3)	295/578 (51·0)	1·14 (0·76–1·70) *P* = 0·533	1·44 (1·04–1·98) *P* = 0·027
Higher/more than secondary	294/952 (30·9)	156/952 (16·4)	502/952 (52·7)	1·17 (0·80–1·72) *P* = 0·410	1·51 (1·12–2·05) *P* = 0·007
Work status (past 12 months)					
Employed	357/1166 (30·6)	234/1166 (20·1)	575/1166 (49·3)	Reference	Reference
Unemployed	336/857 (39·2)	171/857 (20·0)	350/857 (40·8)	0·85 (0·65–1·11) *P* = 0·234	0·75 (0·59–0·94) *P* = 0·012
Other^a^	324/1103 (29·4)	161/1103 (14·6)	618/1103 (56·0)	0·82 (0·57–1·17) *P* = 0·270	0·78 (0·58–1·04) *P* = 0·092
Income tertile					
Low	469/1301 (36·0)	236/1301 (18·1)	596/1301 (45·8)	Reference	Reference
Middle	316/1008 (31·3)	198/1008 (19·6)	494/1008 (49·0)	1·22 (0·96–1·56) *P* = 0·105	1·18 (0·96–1·44) *P* = 0·116
High	232/817 (28·4)	132/817 (16·2)	453/817 (55·4)	1·13 (0·85–1·50) *P* = 0·388	1·38 (1·10–1·72) *P* = 0·005
Country					
Bangladesh	492/1297 (37·9)	281/1297 (21·7)	524/1297 (40·4)	Reference	Reference
India	208/708 (29·4)	124/708 (17·5)	376/708 (53·1)	0·91 (0·68–1·23) *P* = 0·537	1·18 (0·93–1·51) *P* = 0·180
Pakistan	317/1121 (28·3)	161/1121 (14·4)	643/1121 (57·4)	0·75 (0·55–1·03) *P* = 0·074	1·25 (0·97–1·61) *P* = 0·083

Parameter estimates from a multinomial logistic regression model are reported (*n =* 3126).

*Note*: Adjusted estimates extracted from a multinomial logistic regression model including the independent variables: diabetes, hypertension, high cholesterol, high triglycerides, smoking status, physical activity, diet, age group, sex, type of SMI, SMI duration, antipsychotic medication, setting, highest level of education, employment status, income tertile and country.

a Other includes: homemaker, student, and retired.

b Figures in brackets are descriptives which exclude the underweight population and therefore not prevalence data.

RRR, Relative risk ratio; CI, confidence interval.

**Table 4b.** Adjusted associations of comorbidities and health-risk behaviours with overweight and obesity, using WHO Asian cut-offs

	Normal weight^a^ (*n* 1017)	Overweight^a^ (*n* 566)	Obesity^a^ (*n* 1543)	Overweight *v.* normal weight RRR 95 % CI) *P*-value	Obese *v.* normal weight RRR (95 % CI) *P*-value
Current smoker					
No	616/1991 (30·9)	335/1991 (16·8)	1040/1991 (52·2)	Reference	Reference
Yes	401/1135 (35·3)	231/1135 (20·4)	503/1135 (44·3)	0·95 (0·75–1·20) *P*=0·650	0·79 (0·65–0·96) *P* = 0·018
Adequate physical activity					
No	488/1613 (30·3)	260/1613 (16·1)	865/1613 (53·6)	Reference	Reference
Yes	529/1513 (35·0)	306/1513 (20·2)	678/1513 (44·8)	1·03 (0·83–1·28) *P* = 0·786	0·79 (0·66–0·94) *P* = 0·009
Meet WHO recommendations for fruit/veg per day					
No	983/2961 (33·2)	535/2961 (18·1)	1443/2961 (48·7)	Reference	Reference
Yes	34/165 (20·6)	31/165 (18·8)	100/165 (60·6)	1·76 (1·05–2·93) *P* = 0·031	2·53 (1·65–3·88) *P* < 0·001
Pre-diabetes (HbA1c 5·7–6·4%)					
No	808/2411 (33·5)	446/2411 (18·5)	1157/2411 (48·0)	Reference	Reference
Yes	209/715 (29·2)	120/715 (16·8)	386/715 (54·0)	0·94 (0·72–1·22) *P* = 0·642	1·31 (1·06–1·62) *P* = 0·012
Type 2 Diabetes (HbA1c ≥6·5%)					
No	947/2797 (33·9)	515/2797 (18·4)	1335/2797 (47·7)	Reference	Reference
Yes	70/329 (21·3)	51/329 (15·5)	208/329 (63·2)	1·06 (0·71–1·58) *P* = 0·791	1·55 (1·13–2·13) *P* = 0·007
Hypertension (BP ≥140/90 mmHg)					
No	911/2618 (34·8)	491/2618 (18·8)	1216/2618 (46·4)	Reference	Reference
Yes	106/508 (20·9)	75/508 (14·8)	327/508 (64·4)	1·20 (0·86–1·68) *P* = 0·291	1·69 (1·30–2·19) *P* < 0·001
High cholesterol (LDL ≥1 g/l)					
No	572/1527 (37·5)	281/1527 (18·4)	674/1527 (44·1)	Reference	Reference
Yes	445/1599 (27·8)	285/1599 (17·8)	869/1599 (54·3)	1·20 (0·97–1·48) *P* = 0·093	1·32 (1·11–1·56) *P* = 0·002
High triglycerides (≥1.8 g/l)					
No	706/1896 (37·2)	335/1896 (17·7)	855/1896 (45·1)	Reference	Reference
Yes	311/1230 (25·3)	231/1230 (18·8)	688/1230 (55·9)	1·56 (1·25–1·94) *P* < 0·001	1·94 (1·62–2·33) *P* < 0·001

Parameter estimates from a multinomial logistic regression model are reported (*n* = 3126).

*Note*: Adjusted estimates extracted from a multinomial logistic regression model including the independent variables: diabetes, hypertension, high cholesterol, high triglycerides, smoking status, physical activity, diet, age group, sex, type of SMI, SMI duration, antipsychotic medication, setting, highest level of education, employment status, income tertile and country.

a Figures in brackets are descriptives which exclude the underweight population and therefore not prevalence data.

RRR, relative risk ratio; CI, confidence interval.

The relative risk of having obesity (compared with normal weight) is double in women compared with men (RRR = 2·04 [95 % CI: 1·56–2·67]). The percentage of participants with overweight was lower in females compared to males, whilst the percentage of participants with obesity was higher in females compared to males. The percentage of patients with overweight or obesity was higher in females than in males (males 1192/1860 (64·1 %)); females 917/1266 (72·4 %)). Compared to 18–24-year-olds, the 40–54-year age group has the greatest relative risk of having obesity (RRR = 2·91 [95 % CI: 2·15–3·92]), and the relative risk of having obesity increased with longer SMI duration; however, the relative risk of having overweight remained consistent with increasing SMI duration. In participants who were taking antipsychotic medication, the relative risk of having obesity was nearly twice that of participants who were not on medication (RRR = 1·93 [95 % CI: 1·17–3·19]). Participants with higher education, and those in the highest income tertile had the greatest relative risk of obesity compared to no formal education (RRR = 1·51 [95 % CI: 1·12–2·05]) and the lowest income tertile (RRR = 1·38 [95 % CI: 1·10–1·72]), respectively.

The relative risk of having obesity in current smokers was lower (RRR = 0·79 [95 % CI: 0·65–0·96]) than in non-smokers, but the relative risk of being overweight did not differ by smoking status (Table 4b). Participants meeting WHO recommendations for physical activity had 21 % lower risk of having obesity than the less physically active group (RRR = 0·79 [95 % CI: 0·66–0·94]). In contrast, participants who met the WHO recommendations for fruit and vegetable intake had 2·53 (95 % CI: 1·65–3·88) times greater risk of having obesity compared to those not meeting the recommendations.

Participants with pre-diabetes, type 2 diabetes, hypertension, high cholesterol and high triglycerides all had an increased relative risk of having obesity compared to normal weight (Table 4b). The largest relative risk ratio for obesity was seen in participants with hypertriglyceridaemia (RRR = 1·94 [95 % CI: 1·62–2·33]), and this was the only comorbidity for which the relative risk ratio of being overweight was also significant.

In separate models, interaction terms between country and each variable were included. Only age group (*P* = 0·002) and high triglycerides (*P* = 0·048) were identified as having significant interactions with country.

### Association of determinants with abdominal obesity

As seen in Table 5 the odds of having abdominal obesity were 3·79 (95 % CI: 2·99–4·80) higher in women compared with men, and the odds increased with increasing age. With longer SMI duration, there were greater odds of having abdominal obesity, and in those on antipsychotic medication, there was 1·64 (95 % CI: 1·05–2·56) times greater odds of having obesity. Participants in the highest income tertile had 1·26 (95 % CI: 1·04–1·54) times greater odds of having abdominal obesity compared to those in the lowest tertile. Both smoking (OR = 0·89 [95 % CI: 0·75–1·06]) and physical activity (OR = 1·03 [95 % CI: 0·88–1·20]) did not significantly affect the odds of having abdominal obesity in this population; however, participants who ate at least 5 portions of fruit or vegetables per day had 2·35 (95 % CI: 1·65–3·36) times greater odds of abdominal obesity. Participants with type 2 diabetes, hypertension, high cholesterol, and high triglycerides all had higher odds of having abdominal obesity, while no association was found with pre-diabetes.

**Table 5.** Adjusted associations of sociodemographic variables, comorbidities, and health-risk behaviours with abdominal obesity

	Has abdominal obesity (*n* 1767)	No abdominal obesity (*n* 1622)	OR (95 % CI)	*P*-value
Sex				
Male	807/2029 (39·8)	1222/2029 (60·2)	Reference	
Female	960/1360 (70·6)	400/1360 (29·4)	3·79 (2·99–4·80)	*P* < 0·001
Age group				
18–24 years	216/642 (33·6)	426/642 (66·4)	Reference	
25–39 years	809/1598 (50·6)	789/1598 (49·4)	1·87 (1·50–2·33)	*P* < 0·001
40–54 years	539/861 (62·6)	322/861 (37·4)	2·42 (1·86–3·16)	*P* < 0·001
55+ years	203/288 (70·5)	85/288 (29·5)	3·14 (2·18–4·54)	*P* < 0·001
SMI diagnosis				
Bipolar disorder	681/1238 (55·0)	557/1238 (45·0)	Reference	
Major depressive disorder with psychotic features	390/669 (58·3)	279/669 (41·7)	0·76 (0·60–0·96)	*P* = 0·023
Schizophrenia-type disorder	696/1482 (47·0)	786/1482 (53·0)	0·76 (0·63–0·91)	*P* = 0·003
SMI duration				
<2 years	355/799 (44·4)	444/799 (55·6)	Reference	
3–5 years	440/906 (48·6)	466/906 (51·4)	1·25 (1·00–1·55)	*P* = 0·045
6–10 years	436/810 (53·8)	374/810 (46·2)	1·34 (1·06–1·68)	*P* = 0·012
>10 years	536/874 (61·3)	338/874 (38·7)	1·46 (1·15–1·85)	*P* = 0·002
Antipsychotic medication				
No	43/104 (41·3)	61/104 (58·7)	Reference	
Yes	1724/3285 (52·5)	1561/3285 (47·5)	1·64 (1·05–2·56)	*P* = 0·030
Setting				
Inpatient	232/527 (44·0)	295/527 (56·0)	Reference	
Outpatient	1535/2862 (53·6)	1327/2862 (46·4)	1·27 (1·02–1·58)	*P* = 0·029
Level of education				
No formal education	286/472 (60·6)	186/472 (39·4)	Reference	
Primary education	610/1265 (48·2)	655/1265 (51·8)	1·07 (0·82–1·38)	*P* = 0·620
Secondary education	313/624 (50·2)	311/624 (49·8)	1·12 (0·84–1·49)	*P* = 0·436
Higher/more than Secondary	558/1028 (54·3)	470/1028 (45·7)	1·31 (1·00–1·71)	*P* = 0·051
Work status (past 12 months)				
Employed	577/1243 (46·4)	666/1243 (53·6)	Reference	
Unemployed	383/968 (39·6)	585/968 (60·4)	0·83 (0·68–1·01)	*P* = 0·066
Other^a^	807/1178 (68·5)	371/1178 (31·5)	1·12 (0·86–1·45)	*P* = 0·394
Income tertile				
Low	687/1439 (47·7)	752/1439 (52·3)	Reference	
Middle	578/1074 (53·8)	496/1074 (46·2)	1·20 (1·00–1·43)	*P* = 0·050
High	502/876 (57·3)	374/876 (42·7)	1·26 (1·04–1·54)	*P* = 0·019
Country				
Bangladesh	604/1421 (42·5)	817/1421 (57·5)	Reference	
India	449/746 (60·2)	297/746 (39·8)	1·61 (1·30–2·00)	*P* < 0·001
Pakistan	714/1222 (58·4)	508/1222 (41·6)	1·48 (1·19–1·85)	*P* < 0·001
Current smoker				
No	1212/2146 (56·5)	934/2146 (43·5)	Reference	
Yes	555/1243 (44·7)	688/1243 (55·3)	0·89 (0·75–1·06)	*P* = 0·192
Adequate physical activity				
No	964/1759 (54·8)	795/1759 (45·2)	Reference	
Yes	803/1630 (49·3)	827/1630 (50·7)	1·03 (0·88–1·20)	*P* = 0·745
Meet WHO recommendations for fruit/veg per day				
No	1651/3214 (51·4)	1563/3214 (48·6)	Reference	
Yes	116/175 (66·3)	59/175 (33·7)	2·35 (1·65–3·36)	*P* < 0·001
Pre-diabetes (HbA1c 5·7–6·4%)				
No	1364/2632 (51·8)	1268/2632 (48·2)	Reference	
Yes	403/757 (53·2)	354/757 (46·8)	1·06 (0·88–1·28)	*P* = 0·539
Type 2 Diabetes (HbA1c ≥6·5%)				
No	1532/3045 (50·3)	1513/3045 (49·7)	Reference	
Yes	235/344 (68·3)	109/344 (31·7)	1·33 (1·01–1·76)	*P* = 0·041
Hypertension (BP ≥140/90 mmHg)				
No	1404/2863 (49·0)	1459/2863 (51·0)	Reference	
Yes	363/526 (69·0)	163/526 (31·0)	1·69 (1·35–2·11)	*P* < 0·001
High cholesterol (LDL ≥1 g/l)				
No	807/1683 (48·0)	876/1683 (52·0)	Reference	
Yes	960/1706 (56·3)	746/1706 (43·7)	1·17 (1·00–1·36)	*P* = 0·045
High triglycerides (≥1.8 g/l)				
No	1016/2097 (48·5)	1081/2097 (51·5)	Reference	
Yes	751/1292 (58·1)	541/1292 (41·9)	1·69 (1·44–1·98)	*P* < 0·001

Parameter estimates from a logistic regression model are reported (*n* = 3389).

*Note*: Adjusted estimates extracted from a logistic regression model including the independent variables: diabetes, hypertension, high cholesterol, high triglycerides, smoking status, physical activity, diet, age group, sex, type of SMI, SMI duration, antipsychotic medication, setting, highest level of education, employment status, income tertile and country.

a Other includes: homemaker, student, and retired.

OR, odds ratio. CI, confidence interval.

Additional models were fitted including an interaction term between country and each variable. When comparing to a model with no interactions, the likelihood ratio test identified variables sex (*P* < 0·001), age group (*P* = 0·002), level of education (*P* = 0·007), work status (*P* < 0·001), income (*P* = 0·003), and high triglycerides (*P* = 0·029) as having a significant interaction effect with country.

## Discussion

Obesity is a major public health problem in people with SMI regardless of whether international or Asian-specific thresholds for obesity are used, especially considering its association with other chronic conditions. The prevalence of obesity varied according to SMI and the sociodemographic characteristics of participants.

The prevalence of obesity was considerably lower in Bangladesh than in India and Pakistan, which is mirrored in the general population.^(27)^ This may be related to lower income and education in the population in Bangladesh, as explained by other studies.^(28)^ This illustrates the complexity of the interplay between socioeconomic and physical determinants of obesity and how other factors like age may be more influential, as the mean age of people with SMI was lowest in Bangladesh.^(28)^

Although the psychiatric inpatient setting is considered obesogenic,^(29)^ participants in the outpatient setting were more likely to have obesity. Inpatients are more likely to have a refractory degree of SMI which leads to more severe symptoms like catatonia leading to malnutrition.^(30)^ This absence of physical activity can lead to a reduction in bone and muscle density which has been associated with underweight.^(31)^

The higher risk of obesity in women is in line with global trends and is likely driven by socio-cultural factors such as urbanisation where there has been a clear shift in LMICs from agricultural labour to wage labour which is usually more sedentary.^(32)^ The persistent disparity in male and female employment rates, however, shows that women are still more likely to be unemployed and usually in household roles which negatively affects the physical activity of women more than men.^(32,33)^ Clinical studies show that females on antipsychotic medication gain more weight than males.^(34,35)^ Furthermore, on a physiological level, women are more susceptible to weight gain due to their fat distribution and their neural responses to food-related stimuli are more positively correlated with BMI.^(36)^

Contrary to high income countries (HICs) where the poorest are at higher risk of obesity due to poor diet and unhealthy lifestyle,^(37)^ we found the more affluent have an increased risk of obesity. Poorer people in LMICs tend to be engaged in more manual and physically demanding labour which causes increased energy expenditure.^(38)^ Also, our study found that those with a higher level of education are more at risk of being obese compared to no formal education, which may show that food literacy does not correlate with educational attainment. Research has shown that in more developed countries, education can offset the obesogenic effects of increased wealth; however, in LMICs, no interaction was seen between these factors, and both were in fact independent and positively correlated with BMI.^(39)^ This shows that in HICs the more affluent are more likely to purchase better quality and healthier foods, whereas in LMICs the wealthiest have more access to all foods and may gain more weight.^(40)^ This is supported by the finding that participants who met WHO recommendations of fruit and vegetable intake had more than double the risk of obesity compared to those not following this guidance. It is likely that those that can afford to buy and eat more than five fruits or vegetables a day are also the people that can afford more food which increases the relative risk of obesity.^(28)^ The three countries are highly dependent on cereal diets, in the public distribution systems cereals are available at below the market price which in turn increases the consumption of carbohydrates, especially in people from low socioeconomic status.^(41)^ So, although greater vegetable consumption is generally associated with better health outcomes, this should be taken in the context of the whole diet rather than its individual components. Further research should investigate all dietary components.

The results show that people with bipolar disorder are at greatest risk of obesity, which is mirrored in global literature, where the SMI subgroup with the highest prevalence of obesity is bipolar disorder.^(4)^ This is possibly because people with bipolar disorder can experience periods of severe depression which are associated with weight gain, similar to those with major depressive disorder, however they are also likely to experience obesogenic side effects from antipsychotic medication and mood stabilisers.^(42)^

Similar to the general population, obesity was associated with higher relative risk of diabetes, hypertension, high cholesterol, and hypertriglyceridaemia. These are all considered the key features of metabolic syndrome which is associated with three times greater risk of cardiovascular disease and five times greater risk of developing type 2 diabetes,^(22)^ explaining why people with SMI have a 53 % higher risk of developing cardiovascular disease.^(22,43)^ Obesity was associated with hyertriglyceridaemia, which is considered the hallmark of dyslipidaemia and possibly the major cause of all other lipid abnormalities seen in this BMI range.^(44)^ Better screening of lipid abnormalities in people with SMI is required to identify those at risk of dyslipidaemia in this population. Also, obesity was associated with pre-diabetes, which supports the theory of obesity being a strong determinant of pre-diabetes due to the vital role of adipose tissue in systemic insulin resistance.^(45)^

As in the general population, smoking is likely to decrease the risk of obesity due to the appetite suppressing effects of nicotine.^(46,47)^ However, chronic smoking is still considered an important modifiable risk factor with regards to the excess mortality of people with SMI due to its impact on the cardiovascular system through atherosclerosis and the increased risk of lung cancer and chronic obstructive pulmonary disease.^(6,48)^ Smoking cessation interventions used in HICs have been less successful in LMICs hence the urgent need for culturally relevant interventions to be developed.^(49)^

### Strengths and limitations

There are limitations that deserve further attention. First, due to the cross-sectional nature of the study, it is not possible to determine the causality of the associations. Second, we found there was a lack of standardisation in the HbA1c laboratory analysis across the sites; although we used a laboratory in each country which was used in routine clinical practice. Further research is needed to investigate the complexities of HbA1c measurement and show how it affects the prevalence of diabetes across these countries. Third, there was considerable variability in the classification of SMI diagnosis across the different countries, and we found that far more people were diagnosed with major depression with psychotic features using the MINI v6.0. We cross-analysed this with the self-reported diagnoses and found that the vast majority were matching, which suggests that assessor error was unlikely. Fourth, the sample was exclusively from a tertiary centre cohort rather than from the community which may have implications for the interpretation of the results. However, a community survey would be prohibitively resource intensive; moreover, patients at these centres are likely to be similar to those in community, primary or secondary care, as they serve as ‘walk in’ and first point of access services, in the absence of any community mental healthcare.

Despite these limitations, the cross-sectional survey spanned three countries and recruited nearly 4000 participants providing good levels of precision for a population that is often neglected in this area of research. The study also included participants with all forms of SMI, providing evidence about the prevalence of obesity in each type of severe mental disorder. By including analyses using the Asian cut-offs for BMI, our results are easily comparable to other literature in South Asia.

## Conclusion

There is a high prevalence of obesity in the SMI population in Bangladesh, India, and Pakistan. Obesity was associated with chronic disease in this population and, contrary to HICs, people with higher income and higher levels of educational attainment were at greater risk. Food literacy may not correlate with healthier dietary choices and so better dietary education should be prioritised for people with SMI from all levels of educational attainment. People with SMI and obesity could benefit from screening programmes for non-communicable diseases and context appropriate lifestyle interventions to prevent and treat obesity. We have identified the population at higher risk of obesity which provides useful information for intervention development; however, more research is required to identify key barriers for a healthy lifestyle in this population.

## Supporting information

Appuhamy et al. supplementary material Appuhamy et al. supplementary material

## Supplementary material

The supplementary material for this article can be found at https://doi.org/10.1017/jns.2023.100.
