# Supplementary material for: Risk factors associated with overweight and obesity in people with severe mental illness in South Asia: cross-sectional study in Bangladesh, India, and Pakistan
Source: J Nutr Sci. 2023 Nov 21;12:e116. doi: 10.1017/jns.2023.100 (PMC10687724; doi:10.1017/jns.2023.100)
Supplement: Appuhamy et al. supplementary material [file S2048679023001003sup001.docx]

## Appendix

## Participant Flowchart
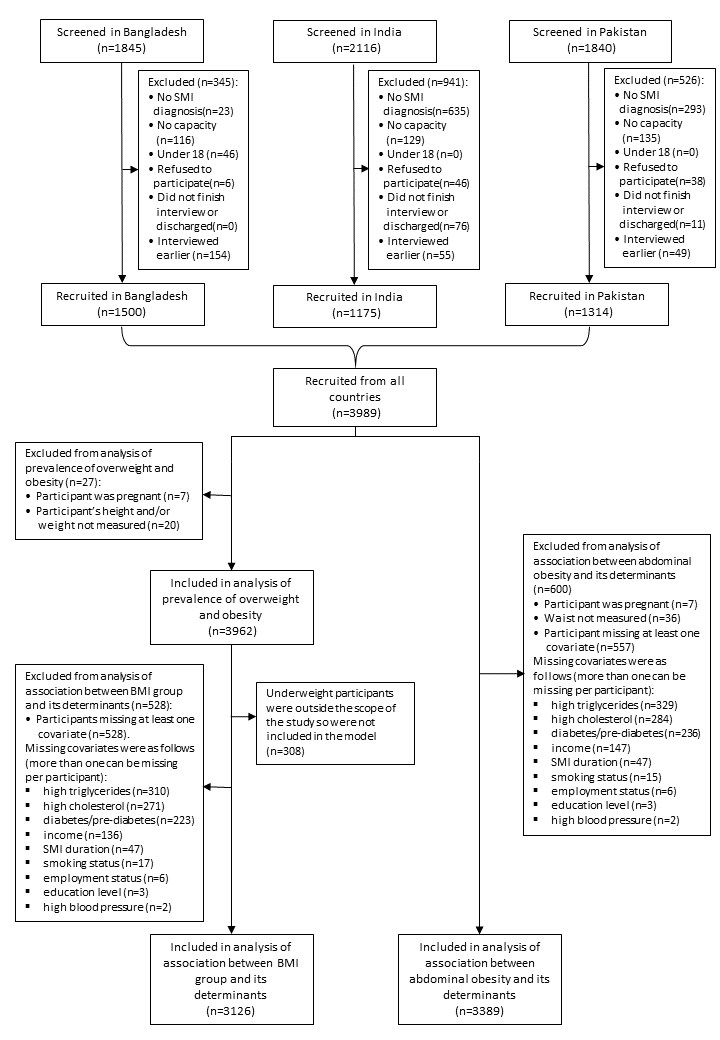


**Figure A1:** Flowchart of participants screened, recruited, and analysed.

## Unadjusted estimates

### Testing the association of determinants of overweight and obesity – unadjusted estimates

**Table A1:** Association of sociodemographic variables, comorbidities and health risk behaviours with overweight and obesity. Unadjusted parameter estimates from multinomial logistic regression models are reported (n=3126).

|  | **Normal weight**  **(n=1017)** | **Overweight**  **(n=566)** | **Obesity**  **(n=1543)** | **Overweight vs. Normal weight**  **RRR (95% CI)**  **p-value** | **Obese vs. Normal weight**  **RRR (95% CI)**  **p-value** |
| --- | --- | --- | --- | --- | --- |
| **Sex** |  |  |  |  |  |
| Male | 668/1860 (35.9) | 380/1860 (20.4) | 812/1860 (43.7) | Reference | Reference |
| Female | 349/1266 (27.6) | 186/1266 (14.7) | 731/1266 (57.7) | 0.94 (0.75-1.17) p=0.558 | 1.72 (1.46-2.03) p<0.001 |
| **Age group** |  |  |  |  |  |
| 18-24 years | 299/567 (52.7) | 100/567 (17.6) | 168/567 (29.6) | Reference | Reference |
| 25-39 years | 452/1484 (30.5) | 284/1484 (19.1) | 748/1484 (50.4) | 1.88 (1.43-2.46) p<0.001 | 2.95 (2.36-3.68) p<0.001 |
| 40-54 years | 188/811 (23.2) | 138/811 (17.0) | 485/811 (59.8) | 2.19 (1.60-3.01) p<0.001 | 4.59 (3.56-5.91) p<0.001 |
| 55+ years | 78/264 (29.5) | 44/264  (16.7) | 142/264 (53.8) | 1.69 (1.09-2.60) p=0.018 | 3.24 (2.32-4.53) p<0.001 |
| **SMI diagnosis** |  |  |  |  |  |
| Bipolar disorder | 346/1171 (29.5) | 201/1171 (17.2) | 624/1171 (53.3) | Reference | Reference |
| Major depressive  disorder with  psychotic features | 180/609 (29.6) | 91/609  (14.9) | 338/609 (55.5) | 0.87 (0.64-1.18) p=0.374 | 1.04 (0.83-1.30) p=0.723 |
| Psychotic disorder | 491/1346 (36.5) | 274/1346 (20.4) | 581/1346 (43.2) | 0.96 (0.76-1.21) p=0.730 | 0.66 (0.55-0.78) p<0.001 |
| **SMI duration** |  |  |  |  |  |
| <2 years | 297/720 (41.3) | 120/720 (16.7) | 303/720 (42.1) | Reference | Reference |
| 3-5 years | 296/832 (35.6) | 170/832 (20.4) | 366/832 (44.0) | 1.42 (1.07-1.89) p=0.015 | 1.21 (0.97-1.51) p=0.089 |
| 6-10 years | 232/755 (30.7) | 146/755 (19.3) | 377/755 (49.9) | 1.56 (1.16-2.09) p=0.003 | 1.59 (1.27-2.00) p<0.001 |
| >10 years | 192/819 (23.4) | 130/819 (15.9) | 497/819 (60.7) | 1.68 (1.23-2.28) p<0.001 | 2.54 (2.01-3.20) p<0.001 |
| **Antipsychotic medication** |  |  |  |  |  |
| No | 42/90 (46.7) | 15/90  (16.7) | 33/90  (36.7) | Reference | Reference |
| Yes | 975/3036 (32.1) | 551/3036 (18.1) | 1510/3036 (49.7) | 1.58 (0.87-2.88) p=0.133 | 1.97 (1.24-3.13) p=0.004 |
| **Setting** |  |  |  |  |  |
| Inpatient | 212/486 (43.6) | 84/486  (17.3) | 190/486 (39.1) | Reference | Reference |
| Outpatient | 805/2640 (30.5) | 482/2640 (18.3) | 1353/2640 (51.3) | 1.51 (1.15-1.99) p=0.003 | 1.88 (1.51-2.32) p<0.001 |
| **Level of education** |  |  |  |  |  |
| No formal education | 132/423 (31.2) | 71/423  (16.8) | 220/423 (52.0) | Reference | Reference |
| Primary education | 408/1173 (34.8) | 239/1173 (20.4) | 526/1173 (44.8) | 1.09 (0.78-1.51) p=0.612 | 0.77 (0.60-0.99) p=0.045 |
| Secondary  education | 183/578 (31.7) | 100/578 (17.3) | 295/578 (51.0) | 1.02 (0.70-1.48) p=0.935 | 0.97 (0.73-1.28) p=0.818 |
| Higher/more than  secondary | 294/952 (30.9) | 156/952 (16.4) | 502/952 (52.7) | 0.99 (0.70-1.40) p=0.939 | 1.02 (0.79-1.33) p=0.855 |
| **Work status**  **(past 12 months)** |  |  |  |  |  |
| Employed | 357/1166 (30.6) | 234/1166 (20.1) | 575/1166 (49.3) | Reference | Reference |
| Unemployed | 336/857 (39.2) | 171/857 (20.0) | 350/857 (40.8) | 0.78 (0.61-0.99) p=0.045 | 0.65 (0.53-0.79) p<0.001 |
| Other^1^ | 324/1103 (29.4) | 161/1103 (14.6) | 618/1103 (56.0) | 0.76 (0.59-0.97) p=0.030 | 1.18 (0.98-1.43) p=0.079 |
| **Income tertile** |  |  |  |  |  |
| Low | 469/1301 (36.0) | 236/1301 (18.1) | 596/1301 (45.8) | Reference | Reference |
| Middle | 316/1008 (31.3) | 198/1008 (19.6) | 494/1008 (49.0) | 1.25 (0.98-1.58) p=0.069 | 1.23 (1.02-1.48) p=0.029 |
| High | 232/817 (28.4) | 132/817 (16.2) | 453/817 (55.4) | 1.13 (0.87-1.47) p=0.363 | 1.54 (1.26-1.88) p<0.001 |
| **Country** |  |  |  |  |  |
| Bangladesh | 492/1297 (37.9) | 281/1297 (21.7) | 524/1297 (40.4) | Reference | Reference |
| India | 208/708 (29.4) | 124/708 (17.5) | 376/708 (53.1) | 1.04 (0.80-1.36) p=0.752 | 1.70 (1.38-2.09) p<0.001 |
| Pakistan | 317/1121 (28.3) | 161/1121 (14.4) | 643/1121 (57.4) | 0.89 (0.70-1.13) p=0.337 | 1.90 (1.59-2.29) p<0.001 |
| **Current smoker** |  |  |  |  |  |
| No | 616/1991 (30.9) | 335/1991 (16.8) | 1040/1991 (52.2) | Reference | Reference |
| Yes | 401/1135 (35.3) | 231/1135 (20.4) | 503/1135 (44.3) | 1.06 (0.86-1.31) p=0.590 | 0.74 (0.63-0.88) p<0.001 |
| **Adequate physical activity** |  |  |  |  |  |
| No | 488/1613 (30.3) | 260/1613 (16.1) | 865/1613 (53.6) | Reference | Reference |
| Yes | 529/1513 (35.0) | 306/1513 (20.2) | 678/1513 (44.8) | 1.09 (0.88-1.33) p=0.434 | 0.72 (0.62-0.85) p<0.001 |
| **Meet WHO recommendations for fruit/veg per day** |  |  |  |  |  |
| No | 983/2961 (33.2) | 535/2961 (18.1) | 1443/2961 (48.7) | Reference | Reference |
| Yes | 34/165 (20.6) | 31/165 (18.8) | 100/165 (60.6) | 1.68 (1.02-2.76) p=0.042 | 2.00 (1.35-2.98) p<0.001 |
| **Pre-diabetes**  **(HbA1c 5.7-6.4)** |  |  |  |  |  |
| No | 808/2411 (33.5) | 446/2411 (18.5) | 1157/2411 (48.0) | Reference | Reference |
| Yes | 209/715 (29.2) | 120/715 (16.8) | 386/715 (54.0) | 1.04 (0.81-1.34) p=0.760 | 1.29 (1.07-1.56) p=0.009 |
| **Type 2 Diabetes**  **(HbA1c ≥ 6.5)** |  |  |  |  |  |
| No | 947/2797 (33.9) | 515/2797 (18.4) | 1335/2797 (47.7) | Reference | Reference |
| Yes | 70/329 (21.3) | 51/329 (15.5) | 208/329 (63.2) | 1.34 (0.92-1.95) p=0.128 | 2.11 (1.59-2.80) p<0.001 |
| **Hypertension**  **(BP ≥ 140/90)** |  |  |  |  |  |
| No | 911/2618 (34.8) | 491/2618 (18.8) | 1216/2618 (46.4) | Reference | Reference |
| Yes | 106/508 (20.9) | 75/508 (14.8) | 327/508 (64.4) | 1.31 (0.96-1.80) p=0.091 | 2.31 (1.83-2.92) p<0.001 |
| **High cholesterol (LDL ≥ 100mg/dL)** |  |  |  |  |  |
| No | 572/1527 (37.5) | 281/1527 (18.4) | 674/1527 (44.1) | Reference | Reference |
| Yes | 445/1599 (27.8) | 285/1599 (17.8) | 869/1599 (54.3) | 1.30 (1.06-1.60) p=0.012 | 1.66 (1.41-1.94) p<0.001 |
| **High triglycerides**  **(≥ 180mg/dL)** |  |  |  |  |  |
| No | 706/1896 (37.2) | 335/1896 (17.7) | 855/1896 (45.1) | Reference | Reference |
| Yes | 311/1230 (25.3) | 231/1230 (18.8) | 688/1230 (55.9) | 1.57 (1.26-1.94) p<0.001 | 1.83 (1.55-2.16) p<0.001 |

^1^ Other includes: homemaker, student, and retired. RRR, Relative risk ratio. CI, Confidence interval.

### Testing the association of determinants of abdominal obesity – unadjusted estimates

**Table A2:** Association of sociodemographic variables, comorbidities and health risk behaviours with abdominal obesity. Unadjusted parameter estimates from logistic regression models are reported (n=3389).

|  | **Has abdominal obesity**  **(n=1767)** | **No abdominal obesity**  **(n=1622)** | **OR (95% CI)** | **p-value** |
| --- | --- | --- | --- | --- |
| **Sex** |  |  |  |  |
| Male | 807/2029 (39.8) | 1222/2029 (60.2) | Reference |  |
| Female | 960/1360 (70.6) | 400/1360 (29.4) | 3.63 (3.14-4.21) | p<0.001 |
| **Age group** |  |  |  |  |
| 18-24 years | 216/642 (33.6) | 426/642 (66.4) | Reference |  |
| 25-39 years | 809/1598 (50.6) | 789/1598 (49.4) | 2.02 (1.67-2.45) | p<0.001 |
| 40-54 years | 539/861 (62.6) | 322/861 (37.4) | 3.30 (2.66-4.09) | p<0.001 |
| 55+ years | 203/288 (70.5) | 85/288 (29.5) | 4.71 (3.48-6.37) | p<0.001 |
| **SMI diagnosis** |  |  |  |  |
| Bipolar disorder | 681/1238 (55.0) | 557/1238 (45.0) | Reference |  |
| Major depressive disorder  with psychotic features | 390/669 (58.3) | 279/669 (41.7) | 1.14 (0.95-1.38) | p=0.167 |
| Psychotic disorder | 696/1482 (47.0) | 786/1482 (53.0) | 0.72 (0.62-0.84) | p<0.001 |
| **SMI duration** |  |  |  |  |
| <2 years | 355/799 (44.4) | 444/799 (55.6) | Reference |  |
| 3-5 years | 440/906 (48.6) | 466/906 (51.4) | 1.18 (0.98-1.43) | p=0.088 |
| 6-10 years | 436/810 (53.8) | 374/810 (46.2) | 1.46 (1.20-1.77) | p<0.001 |
| >10 years | 536/874 (61.3) | 338/874 (38.7) | 1.98 (1.63-2.41) | p<0.001 |
| **Antipsychotic medication** |  |  |  |  |
| No | 43/104 (41.3) | 61/104 (58.7) | Reference |  |
| Yes | 1724/3285 (52.5) | 1561/3285 (47.5) | 1.57 (1.05-2.33) | p=0.026 |
| **Setting** |  |  |  |  |
| Inpatient | 232/527 (44.0) | 295/527 (56.0) | Reference |  |
| Outpatient | 1535/2862 (53.6) | 1327/2862 (46.4) | 1.47 (1.22-1.77) | p<0.001 |
| **Level of education** |  |  |  |  |
| No formal education | 286/472 (60.6) | 186/472 (39.4) | Reference |  |
| Primary education | 610/1265 (48.2) | 655/1265 (51.8) | 0.61 (0.49-0.75) | p<0.001 |
| Secondary education | 313/624 (50.2) | 311/624 (49.8) | 0.65 (0.51-0.83) | p<0.001 |
| Higher/more than  secondary | 558/1028 (54.3) | 470/1028 (45.7) | 0.77 (0.62-0.96) | p=0.022 |
| **Work status**  **(past 12 months)** |  |  |  |  |
| Employed | 577/1243 (46.4) | 666/1243 (53.6) | Reference |  |
| Unemployed | 383/968 (39.6) | 585/968 (60.4) | 0.76 (0.64-0.90) | p=0.001 |
| Other^1^ | 807/1178 (68.5) | 371/1178 (31.5) | 2.51 (2.13-2.96) | p<0.001 |
| **Income tertile** |  |  |  |  |
| Low | 687/1439 (47.7) | 752/1439 (52.3) | Reference |  |
| Middle | 578/1074 (53.8) | 496/1074 (46.2) | 1.28 (1.09-1.49) | p=0.003 |
| High | 502/876 (57.3) | 374/876 (42.7) | 1.47 (1.24-1.74) | p<0.001 |
| **Country** |  |  |  |  |
| Bangladesh | 604/1421 (42.5) | 817/1421 (57.5) | Reference |  |
| India | 449/746 (60.2) | 297/746 (39.8) | 2.04 (1.71-2.45) | p<0.001 |
| Pakistan | 714/1222 (58.4) | 508/1222 (41.6) | 1.90 (1.63-2.22) | p<0.001 |
| **Current smoker** |  |  |  |  |
| No | 1212/2146 (56.5) | 934/2146 (43.5) | Reference |  |
| Yes | 555/1243 (44.7) | 688/1243 (55.3) | 0.62 (0.54-0.72) | p<0.001 |
| **Adequate physical activity** |  |  |  |  |
| No | 964/1759 (54.8) | 795/1759 (45.2) | Reference |  |
| Yes | 803/1630 (49.3) | 827/1630 (50.7) | 0.80 (0.70-0.92) | p=0.001 |
| **Meet WHO recommendations for fruit/veg per day** |  |  |  |  |
| No | 1651/3214 (51.4) | 1563/3214 (48.6) | Reference |  |
| Yes | 116/175 (66.3) | 59/175 (33.7) | 1.86 (1.35-2.57) | p<0.001 |
| **Pre-diabetes**  **(HbA1c 5.7-6.4)** |  |  |  |  |
| No | 1364/2632 (51.8) | 1268/2632 (48.2) | Reference |  |
| Yes | 403/757 (53.2) | 354/757 (46.8) | 1.06 (0.90-1.24) | p=0.493 |
| **Type 2 Diabetes**  **(HbA1c ≥ 6.5)** |  |  |  |  |
| No | 1532/3045 (50.3) | 1513/3045 (49.7) | Reference |  |
| Yes | 235/344 (68.3) | 109/344 (31.7) | 2.13 (1.68-2.70) | p<0.001 |
| **Hypertension**  **(BP ≥ 140/90)** |  |  |  |  |
| No | 1404/2863 (49.0) | 1459/2863 (51.0) | Reference |  |
| Yes | 363/526 (69.0) | 163/526 (31.0) | 2.31 (1.90-2.82) | p<0.001 |
| **High cholesterol (LDL ≥ 100mg/dL)** |  |  |  |  |
| No | 807/1683 (48.0) | 876/1683 (52.0) | Reference |  |
| Yes | 960/1706 (56.3) | 746/1706 (43.7) | 1.40 (1.22-1.60) | p<0.001 |
| **High triglycerides**  **(≥ 180mg/dL)** |  |  |  |  |
| No | 1016/2097 (48.5) | 1081/2097 (51.5) | Reference |  |
| Yes | 751/1292 (58.1) | 541/1292 (41.9) | 1.48 (1.28-1.70) | p<0.001 |

^1^ Other includes: homemaker, student, and retired. OR, Odds ratio. CI, Confidence interval.

## Multiple Imputation analysis

### Testing the association of determinants of overweight and obesity – multiple imputation analysis

Multiple imputation (MI) analysis was performed as a sensitivity analysis, to compare results under a departure from the MAR assumption that was assumed for the complete-case analysis. First, an imputation model containing the outcome variable, covariates and auxiliary variables was used to impute missing data using chained equations, for m=10 imputed datasets (imputation diagnostics and results were compared to using a larger number of imputations; m=50, and results were comparable, therefore m=10 was selected to reduce computation time). Next, the same model as used in the main analysis was performed for each of the imputed datasets. Finally, the results from each of the m=10 models were combined using Rubin’s rules (Rubin, 1987). Parameter estimates are presented in Table A3 alongside the complete-case analysis for comparison.

**Table A3:** Association of sociodemographic variables, comorbidities and health risk behaviours with overweight and obesity. Parameter estimates from a multinomial logistic regression model with multiple imputation are compared to the complete-case analysis.

|  | **Complete-case analysis**  **(n=3126)** | | **Multiple Imputation analysis**  **(n=3674)** | |
| --- | --- | --- | --- | --- |
|  | **Overweight vs. Normal weight**  **RRR (95% CI)**  **p-value** | **Obese vs. Normal weight**  **RRR (95% CI)**  **p-value** | **Overweight vs. Normal weight**  **RRR (95% CI)**  **p-value** | **Obese vs. Normal weight**  **RRR (95% CI)**  **p-value** |
| **Sex** |  |  |  |  |
| Male | Reference | Reference | Reference | Reference |
| Female | 1.09 (0.78-1.51) p=0.611 | 2.04 (1.56-2.67) p<0.001 | 0.96 (0.71-1.30) p=0.795 | 1.97 (1.53-2.52) p<0.001 |
| **Age group** |  |  |  |  |
| 18-24 years | Reference | Reference | Reference | Reference |
| 25-39 years | 1.73 (1.29-2.32) p<0.001 | 2.36 (1.84-3.02) p<0.001 | 1.73 (1.32-2.28) p<0.001 | 2.40 (1.90-3.03) p<0.001 |
| 40-54 years | 2.05 (1.43-2.95) p<0.001 | 2.91 (2.15-3.92) p<0.001 | 1.90 (1.35-2.66) p<0.001 | 2.69 (2.03-3.56) p<0.001 |
| 55+ years | 1.59 (0.96-2.61) p=0.070 | 1.63 (1.09-2.44) p=0.018 | 1.52 (0.96-2.39) p=0.072 | 1.41 (0.96-2.05) p=0.078 |
| **SMI diagnosis** |  |  |  |  |
| Bipolar disorder | Reference | Reference | Reference | Reference |
| Major depressive  disorder with  psychotic features | 0.95 (0.67-1.34) p=0.755 | 0.81 (0.63-1.06) p=0.130 | 0.99 (0.71-1.37) p=0.942 | 0.84 (0.65-1.08) p=0.175 |
| Psychotic disorder | 0.91 (0.71-1.17) p=0.462 | 0.69 (0.57-0.85) p<0.001 | 0.92 (0.74-1.16) p=0.490 | 0.70 (0.58-0.84) p<0.001 |
| **SMI duration** |  |  |  |  |
| <2 years | Reference | Reference | Reference | Reference |
| 3-5 years | 1.32 (0.99-1.77) p=0.062 | 1.14 (0.90-1.45) p=0.285 | 1.33 (1.01-1.75) p=0.040 | 1.13 (0.90-1.42) p=0.283 |
| 6-10 years | 1.33 (0.97-1.81) p=0.076 | 1.30 (1.01-1.68) p=0.044 | 1.35 (1.01-1.81) p=0.042 | 1.28 (1.01-1.63) p=0.040 |
| >10 years | 1.36 (0.97-1.91) p=0.077 | 1.76 (1.35-2.31) p<0.001 | 1.41 (1.03-1.92) p=0.032 | 1.66 (1.29-2.14) p<0.001 |
| **Antipsychotic medication** |  |  |  |  |
| No | Reference | Reference | Reference | Reference |
| Yes | 1.36 (0.73-2.51) p=0.333 | 1.93 (1.17-3.19) p=0.010 | 1.10 (0.64-1.90) p=0.720 | 1.75 (1.10-2.79) p=0.019 |
| **Setting** |  |  |  |  |
| Inpatient | Reference | Reference | Reference | Reference |
| Outpatient | 1.42 (1.07-1.90) p=0.017 | 1.57 (1.24-2.00) p<0.001 | 1.40 (1.08-1.82) p=0.010 | 1.52 (1.22-1.88) p<0.001 |
| **Level of education** |  |  |  |  |
| No formal education | Reference | Reference | Reference | Reference |
| Primary education | 1.15 (0.81-1.64) p=0.437 | 1.23 (0.92-1.65) p=0.154 | 1.15 (0.83-1.60) p=0.394 | 1.28 (0.98-1.68) p=0.073 |
| Secondary  education | 1.14 (0.76-1.70) p=0.533 | 1.44 (1.04-1.98) p=0.027 | 1.14 (0.79-1.66) p=0.475 | 1.47 (1.08-1.98) p=0.013 |
| Higher/more than  secondary | 1.17 (0.80-1.72) p=0.410 | 1.51 (1.12-2.05) p=0.007 | 1.12 (0.79-1.60) p=0.518 | 1.61 (1.21-2.15) p=0.001 |
| **Work status**  **(past 12 months)** |  |  |  |  |
| Employed | Reference | Reference | Reference | Reference |
| Unemployed | 0.85 (0.65-1.11) p=0.234 | 0.75 (0.59-0.94) p=0.012 | 0.81 (0.63-1.04) p=0.093 | 0.76 (0.62-0.95) p=0.013 |
| Other^1^ | 0.82 (0.57-1.17) p=0.270 | 0.78 (0.58-1.04) p=0.092 | 0.87 (0.63-1.22) p=0.429 | 0.82 (0.62-1.07) p=0.145 |
| **Income tertile** |  |  |  |  |
| Low | Reference | Reference | Reference | Reference |
| Middle | 1.22 (0.96-1.56) p=0.105 | 1.18 (0.96-1.44) p=0.116 | 1.39 (1.10-1.74) p=0.005 | 1.16 (0.96-1.40) p=0.132 |
| High | 1.13 (0.85-1.50) p=0.388 | 1.38 (1.10-1.72) p=0.005 | 1.25 (0.95-1.63) p=0.105 | 1.43 (1.15-1.78) p=0.001 |
| **Country** |  |  |  |  |
| Bangladesh | Reference | Reference | Reference | Reference |
| India | 0.91 (0.68-1.23) p=0.537 | 1.18 (0.93-1.51) p=0.180 | 1.14 (0.88-1.48) p=0.324 | 1.35 (1.09-1.69) p=0.007 |
| Pakistan | 0.75 (0.55-1.03) p=0.074 | 1.25 (0.97-1.61) p=0.083 | 0.78 (0.57-1.05) p=0.105 | 1.30 (1.02-1.66) p=0.036 |
| **Current smoker** |  |  |  |  |
| No | Reference | Reference | Reference | Reference |
| Yes | 0.95 (0.75-1.20) p=0.650 | 0.79 (0.65-0.96) p=0.018 | 0.92 (0.74-1.15) p=0.469 | 0.82 (0.68-0.99) p=0.035 |
| **Adequate physical activity** |  |  |  |  |
| No | Reference | Reference | Reference | Reference |
| Yes | 1.03 (0.83-1.28) p=0.786 | 0.79 (0.66-0.94) p=0.009 | 1.01 (0.82-1.24) p=0.913 | 0.83 (0.70-0.98) p=0.030 |
| **Meet WHO recommendations for fruit/veg per day** |  |  |  |  |
| No | Reference | Reference | Reference | Reference |
| Yes | 1.76 (1.05-2.93) p=0.031 | 2.53 (1.65-3.88) p<0.001 | 2.18 (1.36-3.51) p=0.001 | 2.47 (1.63-3.74) p<0.001 |
| **Pre-diabetes**  **(HbA1c 5.7-6.4)** |  |  |  |  |
| No | Reference | Reference | Reference | Reference |
| Yes | 0.94 (0.72-1.22) p=0.642 | 1.31 (1.06-1.62) p=0.012 | 0.91 (0.70-1.18) p=0.475 | 1.35 (1.10-1.65) p=0.004 |
| **Type 2 Diabetes**  **(HbA1c ≥ 6.5)** |  |  |  |  |
| No | Reference | Reference | Reference | Reference |
| Yes | 1.06 (0.71-1.58) p=0.791 | 1.55 (1.13-2.13) p=0.007 | 1.12 (0.77-1.62) p=0.563 | 1.75 (1.29-2.37) p<0.001 |
| **Hypertension**  **(BP ≥ 140/90)** |  |  |  |  |
| No | Reference | Reference | Reference | Reference |
| Yes | 1.20 (0.86-1.68) p=0.291 | 1.69 (1.30-2.19) p<0.001 | 1.33 (0.97-1.82) p=0.073 | 1.88 (1.47-2.41) p<0.001 |
| **High cholesterol (LDL ≥ 100mg/dL)** |  |  |  |  |
| No | Reference | Reference | Reference | Reference |
| Yes | 1.20 (0.97-1.48) p=0.093 | 1.32 (1.11-1.56) p=0.002 | 1.19 (0.96-1.47) p=0.112 | 1.33 (1.12-1.59) p=0.001 |
| **High triglycerides**  **(≥ 180mg/dL)** |  |  |  |  |
| No | Reference | Reference | Reference | Reference |
| Yes | 1.56 (1.25-1.94) p<0.001 | 1.94 (1.62-2.33) p<0.001 | 1.61 (1.29-2.00) p<0.001 | 1.93 (1.62-2.30) p<0.001 |

^1^ Other includes: homemaker, student, and retired. RRR, Relative risk ratio. CI, Confidence interval.

### Testing the association of determinants of abdominal obesity – multiple imputation analysis

MI was also performed for the abdominal obesity model following the same steps as described above. Parameter estimates are presented in Table A4 alongside the complete-case analysis for comparison.

**Table A4:** Association of sociodemographic variables, comorbidities and health risk behaviours with abdominal obesity. Parameter estimates from a logistic regression model with multiple imputation are compared to the complete-case analysis.

|  | **Complete-case analysis**  **(n=3389)** | | **Multiple Imputation analysis**  **(n=3982)** | |
| --- | --- | --- | --- | --- |
| **Odds of abdominal obesity** | **OR (95% CI)** | **p-value** | **OR (95% CI)** | **p-value** |
| **Sex** |  |  |  |  |
| Male | Reference |  | Reference |  |
| Female | 3.79 (2.99-4.80) | p<0.001 | 3.70 (2.97-4.60) | p<0.001 |
| **Age group** |  |  |  |  |
| 18-24 years | Reference |  | Reference |  |
| 25-39 years | 1.87 (1.50-2.33) | p<0.001 | 1.77 (1.43-2.18) | p<0.001 |
| 40-54 years | 2.42 (1.86-3.16) | p<0.001 | 2.26 (1.77-2.89) | p<0.001 |
| 55+ years | 3.14 (2.18-4.54) | p<0.001 | 2.77 (1.97-3.90) | p<0.001 |
| **SMI diagnosis** |  |  |  |  |
| Bipolar disorder | Reference |  | Reference |  |
| Major depressive disorder  with psychotic features | 0.76 (0.60-0.96) | p=0.023 | 0.76 (0.61-0.95) | p=0.015 |
| Psychotic disorder | 0.76 (0.63-0.91) | p=0.003 | 0.73 (0.62-0.86) | p<0.001 |
| **SMI duration** |  |  |  |  |
| <2 years | Reference |  | Reference |  |
| 3-5 years | 1.25 (1.00-1.55) | p=0.045 | 1.21 (0.99-1.47) | p=0.062 |
| 6-10 years | 1.34 (1.06-1.68) | p=0.012 | 1.34 (1.08-1.65) | p=0.007 |
| >10 years | 1.46 (1.15-1.85) | p=0.002 | 1.37 (1.10-1.70) | p=0.005 |
| **Antipsychotic medication** |  |  |  |  |
| No | Reference |  | Reference |  |
| Yes | 1.64 (1.05-2.56) | p=0.030 | 1.52 (1.01-2.29) | p=0.046 |
| **Setting** |  |  |  |  |
| Inpatient | Reference |  | Reference |  |
| Outpatient | 1.27 (1.02-1.58) | p=0.029 | 1.31 (1.08-1.59) | p=0.006 |
| **Level of education** |  |  |  |  |
| No formal education | Reference |  | Reference |  |
| Primary education | 1.07 (0.82-1.38) | p=0.620 | 1.18 (0.93-1.50) | p=0.175 |
| Secondary education | 1.12 (0.84-1.49) | p=0.436 | 1.24 (0.95-1.62) | p=0.108 |
| Higher/more than  secondary | 1.31 (1.00-1.71) | p=0.051 | 1.38 (1.07-1.77) | p=0.012 |
| **Work status**  **(past 12 months)** |  |  |  |  |
| Employed | Reference |  | Reference |  |
| Unemployed | 0.83 (0.68-1.01) | p=0.066 | 0.81 (0.68-0.98) | p=0.028 |
| Other^1^ | 1.12 (0.86-1.45) | p=0.394 | 1.10 (0.86-1.39) | p=0.453 |
| **Income tertile** |  |  |  |  |
| Low | Reference |  | Reference |  |
| Middle | 1.20 (1.00-1.43) | p=0.050 | 1.23 (1.04-1.45) | p=0.018 |
| High | 1.26 (1.04-1.54) | p=0.019 | 1.30 (1.08-1.56) | p=0.005 |
| **Country** |  |  |  |  |
| Bangladesh | Reference |  | Reference |  |
| India | 1.61 (1.30-2.00) | p<0.001 | 1.76 (1.46-2.13) | p<0.001 |
| Pakistan | 1.48 (1.19-1.85) | p<0.001 | 1.52 (1.23-1.88) | p<0.001 |
| **Current smoker** |  |  |  |  |
| No | Reference |  | Reference |  |
| Yes | 0.89 (0.75-1.06) | p=0.192 | 0.90 (0.77-1.06) | p=0.219 |
| **Adequate physical activity** |  |  |  |  |
| No | Reference |  | Reference |  |
| Yes | 1.03 (0.88-1.20) | p=0.745 | 1.07 (0.92-1.24) | p=0.365 |
| **Meet WHO recommendations for fruit/veg per day** |  |  |  |  |
| No | Reference |  | Reference |  |
| Yes | 2.35 (1.65-3.36) | p<0.001 | 2.44 (1.74-3.43) | p<0.001 |
| **Pre-diabetes**  **(HbA1c 5.7-6.4)** |  |  |  |  |
| No | Reference |  | Reference |  |
| Yes | 1.06 (0.88-1.28) | p=0.539 | 1.11 (0.93-1.33) | p=0.233 |
| **Type 2 Diabetes**  **(HbA1c ≥ 6.5)** |  |  |  |  |
| No | Reference |  | Reference |  |
| Yes | 1.33 (1.01-1.76) | p=0.041 | 1.55 (1.18-2.03) | p=0.002 |
| **Hypertension**  **(BP ≥ 140/90)** |  |  |  |  |
| No | Reference |  | Reference |  |
| Yes | 1.69 (1.35-2.11) | p<0.001 | 1.69 (1.37-2.09) | p<0.001 |
| **High cholesterol (LDL ≥ 100mg/dL)** |  |  |  |  |
| No | Reference |  | Reference |  |
| Yes | 1.17 (1.00-1.36) | p=0.045 | 1.19 (1.03-1.38) | p=0.017 |
| **High triglycerides**  **(≥ 180mg/dL)** |  |  |  |  |
| No | Reference |  | Reference |  |
| Yes | 1.69 (1.44-1.98) | p<0.001 | 1.73 (1.48-2.01) | p<0.001 |

^1^ Other includes: homemaker, student, and retired. OR, Odds ratio. CI, Confidence interval.
